# Excreted/Secreted 15-kDa Proteins and Serine Protease Peptides from *Haemonchus contortus* Act as Immune-Response Enhancers in Lambs

**DOI:** 10.3390/pathogens13070604

**Published:** 2024-07-22

**Authors:** René Camas-Pereyra, Génesis Andrea Bautista-García, Gustavo Pérez-Anzúrez, Zaira Carolina Duran-Cortes, David Emanuel Reyes-Guerrero, Jocelyn Maza-Lopez, Agustín Olmedo-Juárez, María Eugenia López-Arellano

**Affiliations:** 1 Centro Nacional de Investigación Disciplinaria en Salud Animal e Inocuidad, Instituto Nacional de Investigaciones Forestales, Agrícolas y Pecuarias, Carr. Fed. Cuernavaca-Cuautla 8534, Jiutepec 62574, Mexico; camas.rene@inifap.gob.mx (R.C.-P.); reyes.david@inifap.gob.mx (D.E.R.-G.); olmedo.agustin@inifap.gob.mx (A.O.-J.); 2 Facultad de Medicina Veterinaria y Zootecnia, Ciudad Universitaria, Ciudad de México 04510, Mexico; bagg150583@gmail.com (G.A.B.-G.); tavopzaz@gmail.com (G.P.-A.); jomal1993@gmail.com (J.M.-L.); 3 Ingeniería en Biotecnología, Universidad Politécnica del Estado de Morelos, Boulevard Cuauhnauhuac #566, Jiutepec 62550, Mexico; caroduran04@hotmail.com

**Keywords:** *Haemonchus* spp., S28 peptides, 15kDa protein, cytokines, IgG, vaccine

## Abstract

This study assessed the immunoprotective effect in lambs of a native excreted/secreted 15-kDa protein and two synthesised S28 peptides derived from the infective transitory larvae (xL_3_) and adult stages (AS) of *Haemonchus contortus*. Twenty-two Pelibuey lambs were divided into negative and positive control groups, as well as immunised lamb groups, with 100 µg of the 15-kDa native protein (15kDaNP) and S28 peptides (S28P). The eggs per gram (EPG) and haematocrit were measured, and AS were counted and morphologically measured. To assess the immunoprotection in lambs, indirect enzyme-linked immunosorbent assays and relative expression analyses of immune cytokines were performed using serum and abomasal samples. Our results showed a 72.28% reduction in adult worms (AW) in the 15kDaNP-immunised group, achieving a high clinical response with 41% haematocrit and low EPG values (436 ± 661). Conversely, the S28P group achieved the highest IgG levels (2.125 ± 0.880 OD), with AW exhibiting the greatest body length (*p* > 0.05) and upregulation of the *IL5* and *FCεR1A* genes associated with nematode control. The 15kDaNP group showed increased expression of genes related to nematode control and anti-inflammatory responses, including *IL4*, *IL5*, *IL6*, and *IL13* (*p* < 0.05). The S28P and 15kDaNP should be explored as potential vaccines against sheep haemonchosis.

## 1. Introduction

*Haemonchus contortus* is considered one of the most pathogenic and prevalent species of gastrointestinal nematodes (GINs) of the Trichostrongylidae family in small ruminants worldwide [1]. Its haematophagous habits induce anaemia and hyperproteinaemia in infected hosts, clinically manifesting as a hyperacute, acute, and chronic disease [2]. The high prevalence of *H. contortus* and host susceptibility to haemonchosis pose significant animal-health concerns, leading to severe economic losses and reduced productivity in grazing animals [3]. For decades, haemonchosis has been controlled by administering chemically synthesised anthelmintic drugs. However, indiscriminate use of these drugs has increased the number of resistant nematode populations [4,5] and has negatively impacted the environment by affecting beneficial soil micro-organisms [6,7]. In search of alternatives to chemical anthelmintic drugs, researchers have focused on sustainable and harmless control strategies, such as vaccines [8]. Researchers from Australia developed a native vaccine against *H. contortus*, commercially known as Barbervax (Wormvax Australia Pty Ltd., Albany, Australia) [9]. The antigens used to achieve the immunoprotection in this vaccine were derived from a set of intestinal hidden antigens called H-gal-GP and H11 [10,11]. This vaccine is commercially available in Australia, the United Kingdom, and South Africa (https://barbervax.com/14 accessed on 1 July 2024). Studies assessing the level of immunoprotection induced by this vaccine against *H. contortus* reported 90% reduction in the faecal egg count [12,13] and a 70% to 80% reduction in adult parasite numbers [10].

Researchers are working to improve the efficacy of this vaccine by exploring other immunogenic antigens such as serine proteases [14], metalloproteases [15], galectins [16], and glycoproteins [17], as well as using co-adjuvants such as Con A-purified proteins [18]. Additionally, nutritional strategies to enhance the immune response in flocks are currently under study [19,20,21]. Various antigens with immunoprotective activity against *H. contortus* and other GINs have been explored. These include hidden and surface antigens [22,23,24], as well as excreted/secreted (E/S) proteins that activate both humoral and cellular immune responses against *H. contortus* and *Teladorsagia circumcincta* [25,26].

E/S proteins are involved in diverse biological functions at the host–parasite interface, some of which are associated with proteases from the gut surface of *Haemonchus* sp. and other helminths [27,28,29]. During the pathogenicity of *H. contortus*, these E/S proteins are exposed to the host’s immune system as the infective larvae invade host tissue and acquire their blood-feeding habit to reach the adult stage [30,31,32]. The resultant tissue damage triggers the host’s humoral and cell-mediated immune responses, which involve inflammatory and regulatory mechanisms aimed at reducing the nematode infection [33,34]. Beyond this, proteomic and genomic studies have explored various functional peptides from E/S products and related intestinal proteins from the adult stages of *H. contortus*. These studies have identified potential vaccines against this parasite [35,36]. In this context, our research group has focused special attention on exploring the immune response of a 15-kDa E/S protein collected from *Haemonchus* spp. transitory larvae (xL_3_) and two synthetic peptides from the serine-type protease family (S28) that have shown immunogenic activity associated with T_H_1 and T_H_2 immune cells [28,29]. However, it is necessary to evaluate whether the findings observed under in vitro conditions can be used for comparison in an in vivo study of the immune response in immunised and challenged young sheep. Therefore, the present study was performed to evaluate the immunoprotective effect of a 15-kDa native *H. contortus* E/S protein and two synthesised S28 peptides derived from the transitory larvae (xL_3_) and adult stages of *H. contortus* in immunised and challenged Pelibuey lambs.

## 2. Materials and Methods

### 2.1. Haemonchus Contortus Donor Sheep

A 6-month-old male sheep was used as the parasite donor. It was kept in an individual pen at the National Center for Disciplinary Research in Animal Health and Innocuity (CENID-SAI-INIFAP) in Jiutepec municipality, Morelos, Mexico. This animal was artificially infected (per os) with 10,000 L_3_ of *H. contortus*. After a 21-day pre-patent period, faeces were collected directly from the rectum of the animal. The faeces were processed to produce faecal cultures according to the method described by Figueroa-Castillo et al. (2015) [37]. Infective larvae were recovered from the faecal cultures using the Baermann funnel technique. The recovered larvae were washed by density gradient centrifugation using 40% sucrose, followed by rinsing with sterile distilled water to remove any sucrose residues. The clean larvae were unsheathed using 0.187% sodium hypochlorite solution. The unsheathed larvae were rinsed three times with sterile distilled water to remove the larval sheaths. Centrifugation was performed three times at 1000× *g* for 1 min at room temperature (18–25 °C) [38].

### 2.2. Synthesis of S28 Peptides from Haemonchus spp. Adult Stages

Following the methods described in a previous study [29], two S28 peptides were synthesised: pep-pcx + pep-hsp. Briefly, the synthesis involved a system of linear peptides for indirect enzyme-linked immunosorbent assays (iELISAs) and multiple antigenic peptides with eight branches (MAP-8) for immunisations. Before use, the linear and MAP-8 fractions were solubilised in phosphate-buffered saline (PBS) at pH 7.4, and sonicated using a Cole-Parmer ultrasonic cleaner at a frequency of 40 kHz and temperature of 4 °C for 15 to 30 min. Prior to immunisation, each peptide fraction was quantified using the Qubit method in accordance with the manufacturer’s instructions (Invitrogen Assay, ThermoFisher, Waltham, MA, USA).

### 2.3. Native 15-kDa E/S Protein from H. contortus Transitory Larvae (xL_3_)

*Haemonchus contortus* unsheathed larvae (3000–4000 L_3_) were incubated with PBS (pH 7.2) and supplemented with an antibiotic/antifungal (penicillin 10,000 IU/mL, streptomycin 10,000 µg/mL, and amphotericin 25 µg/mL 100×) (Invitrogen Assay, CA, USA) overnight at room temperature (21–32 °C). The larvae were incubated in Hank’s culture medium at 37 °C with 5% carbon dioxide in 25 cm^3^ cell culture boxes (Thermo Fisher Scientific, Waltham, MA, USA). At 24, 48, and 72 h and at 5 and 7 days, media from each culture plate were collected to obtain the xL_3_ E/S proteins. The same volume of recovered medium was replaced by a new sterile medium. The samples were concentrated with 5-kDa Spin-X^®^ UF tubes (Corning Inc., Corning, NY, USA) and centrifuged at 12,000× *g* for 30 min at 4 °C. The recovered proteins were separated and visualised using sodium dodecyl sulphate polyacrylamide gel electrophoresis with 5% and 12% gels. Total proteins were estimated using the Qubit method (Invitrogen Assay, ThermoFisher, Waltham, MA, USA) [28].

### 2.4. Experimental Design

**Figure 1:**
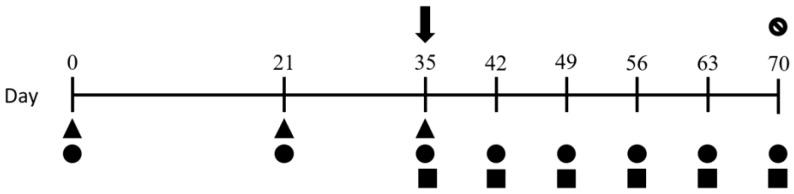
Experimental design. Detailed immunisation calendar and sample collections are indicated as follows: (▲) immunisations, (↓) challenge infection with *H. contortus*, (●) blood sample collection, (■) faecal sample collection, and (ᴓ) lamb slaughter.

Twenty-two 4-month-old gastrointestinal parasite-free Pelibuey lambs were kept in four pens at CENID-SAI-INIFAP. The animals were fed a maintenance diet based on lamb concentrate food, plus trimmed alfalfa and water ad libitum. Four experimental groups were established: negative control (NC) (n = 4), positive control (PC) (n = 6), S28 peptides (pep-pcx + pep-hsp) (S28P) (n = 6), and 15-kDa native protein (15kDaNP) (n = 6). The steps of the whole procedure are summarised in Figure 1. Briefly, 6 mL blood samples were collected from the jugular vein using a BD Vacutainer^®^ blood-transfer device and placed in BD Vacutainer^®^ tubes (Becton, Dickinson and Company, Franklin Lakes, NJ, USA). Capillary micro-tubes were used to determine the haematocrit (Ht). Blood was centrifuged to obtain serum for the iELISA assays. Additionally, a 10 g faecal sample was obtained and processed by the McMaster technique to estimate the number of eggs per gram of faeces (EPG) [39,40].

Negative samples from all experimental sheep were collected on day 0. Three immunisations were then subcutaneously administered near the prescapular nodules on days 0, 21, and 35 of the experiment. The immunising treatment consisted of 70% Montanide ISA 201 VG (SEPPIC, Paris, France) + 30% of the mixed peptide solution/native 15-kDa protein [29]. After the third immunisation on day 35, the lambs were orally challenged with 10,000 L_3_ of *H. contortus*. Clinical signs were monitored weekly until day 70, at which time the lambs were euthanised and necropsied for pathological analysis and parasite counting. Euthanasia was performed in strict accordance with protocols to avoid unnecessary animal suffering as established by the Official Mexican Standard NOM-033-SAG/ZOO-2014, normative Section 5.4.2.1 (https://www.gob.mx/senasica/ accessed on 14 July 2024).

### 2.5. Abomasal Tissues, Adult Worm Recovery, and Morphometry

Abomasal tissue specimens (50 mm^2^) were collected from the fundic region of each animal in the experimental groups. Tissue sections were washed with PBS at pH 7.4 (0.15 M) and preserved in RNAlater Stabilization Solution (QIAGEN, Venlo, The Netherlands) at −20 °C to perform relative expression studies. Both female and male adult parasites were recovered directly from tissue and abomasal content. The parasites were washed and decontaminated with PBS at pH 7.4 (0.15 M, 0.01% sodium azide). The recovered biological material was stored in 70% ethanol at 4 °C. Adult parasites from the immunised groups and PC group were recovered and analysed for possible morphological changes. The recovered nematodes were observed under a light microscope (20× and 40× objective lenses) (LEICA DM6; Leica Microsystems, Wetzlar, Germany), and the total length of the adult parasites, as well as the oesophageal length in both males and females, were measured. Other characteristics were also measured and recorded, including the distance between the vulva and tail, the female tail width, the spicule length, and the gubernaculum width

### 2.6. iELISA

The iELISA technique was performed in 96-well, flat-bottom microplates in accordance with the method described by Camas-Pereyra et al. (2023) [29]. Two types of *H. contortus* antigens were added to the plate with a carbonate buffer (pH 9.6) and incubated at 4 °C overnight: (1) a mixture of two synthetic adult S28 linear peptides at a final concentration of 0.25 μg/mL (pep-pcx 1:1pep-hsp) and (2) a 15-kDa native xL3 E/S protein at 0.625 μg/mL. Serum samples from each experimental day were then analysed per group in accordance with the previously described experimental design. Briefly, serum from each animal in each experimental group was diluted to 1:400, with PBS containing Tween 20 at 0.1%, and added to the wells of the microplate (n = 3). A conjugate, rabbit anti-sheep IgG (H+L) HRP was diluted to 1:7500 with PBS containing Tween 20 at 0.1%. The plate was incubated at room temperature (18–25 °C) for 1 h. Finally, the microplate was read at an optical density (OD) of 450 nm using 3,3′,5,5′-tetramethylbenzidine as the substrate (Sigma-Aldrich, St. Louis, MO, USA) and 2N sulphuric acid as the stop solution. The whole experiment was repeated in triplicate.

### 2.7. RNA Extraction and Reverse Transcription

Total RNA (_T_RNA) was extracted from abomasal tissue samples based on the following RNA extraction protocol (TRIzol^®^, Thermo Fisher Scientific, Waltham, MA, USA). The concentration and purity of _T_RNA were estimated using a NanoPhotometer NP80 (Implen, Munich, Germany). The integrity of _T_RNA was visualised using 1.5% agarose gel electrophoresis, and any DNA contamination was eliminated using a commercial kit (RQ RNase-Free DNase^®^, Promega, Madison, WI, USA). Reverse transcription was then performed to obtain complementary DNA using 300 ng of _T_RNA and a random oligonucleotide in accordance with the manufacturer’s instructions (ImProm-II Reverse Transcription System^®^, Promega, Madison, WI, USA).

### 2.8. Quantitative Polymerase Chain Reaction (qPCR) and Relative Gene Expression

The gene expression of interleukin *(IL)4*, *IL5*, *IL6*, *IL13*, *CXCL8*, and *FCεR1A* was evaluated by qPCR, using housekeeping genes (*β-actin* and *GAPDH*) (Table S1). The reaction was performed in 0.2 mL nuclease-free PCR tubes, with a final volume of 20 μL, containing the commercial product GoTaq^®^ qPCR Master Mix 2× (Promega, Madison, WI, USA) and each pair of oligonucleotides at a concentration of 20 µM.

The qPCR conditions were as follows: initial denaturation at 95 °C for 10 min, cycling of 40 repetitions and denaturation at 95 °C for 15 s, alignment at 60 °C for 20 s, and extension at 72 °C for 20 s. The reverse-transcription qPCR products were processed in a Rotor-Gene Q thermocycler (Corbett 6000 Research, Qiagen, Hilden, Germany). The size of the amplicons was confirmed using 1.5% agarose gel electrophoresis. The dissociation temperatures and accession numbers of the primers used in this study were previously described by Camas-Pereyra et al. (2023) [29].

### 2.9. Statistical Analysis

The data were analysed with SPSS Version 25 (IBM Corp., Armonk, NY, USA). The haematocrit and iELISA values were subjected to analysis of variance followed by a multiple comparison of means using Tukey’s test. The iELISA S28 peptide group data were analysed by Dunnett’s T3 test. The EPG data were analysed by the non-parametric Kruskal–Wallis test, in which all pairs of means were compared. A significance threshold of *p* = 0.05 was used for all assays. The cycle threshold (C_T_) value for each cytokine and receptor gene under study was subtracted from the average C_T_ value of housekeeping genes to normalise the expression, and the resultant value was defined as ΔC_T_. The ΔΔC_T_ average value was then estimated from each group to obtain the fold change per replicate (n = 3), and the statistical data were analysed using Student’s *t* test (*p* = 0.05) on the Qiagen GeneGlobe platform (https://geneglobe.qiagen.com accessed on 1 February 2024). Finally, a Pearson correlation test was performed on the dataset of phenotypic traits (haematocrit, EPG, and IgG levels) and the fold change values from the relative gene expression analysis (*p* < 0.05).

## 3. Results

### 3.1. Haematocrit and Faecal Egg Counts

**Figure 2:**
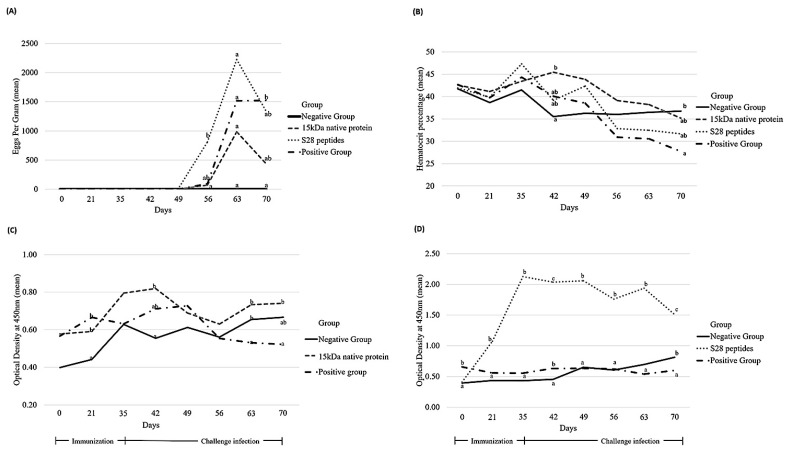
Summary of phenotypic traits evaluated in four experimental lamb groups. (**A**) Eggs per gram of faeces. (**B**) Haematocrit. (**C**,**D**) Dynamics of peripheral IgG observed in treated lambs with the 15-kDa native protein group and the S28 peptide group, respectively. Different literals indicate statistically significant differences. (-·-·) Positive control group, (—) Negative control group, (---) Native 15-kDa protein group, and (····) S28 peptide group.

First, to evaluate the clinical response of *H. contortus*-challenged lambs, the haematocrit was determined from days 0 to 70 as an indicator of anaemia. The haematocrit showed a minimal decrease after day 35 in all experimental groups. The group with the native 15-kDa protein showed the highest haematocrit during the experiment (x = 41%). Significant differences among the groups were observed only after day 42, and a marked difference was observed on day 70 (*p* < 0.05) (Figure 2B).

Next, the EPG value was highest in the S28 peptide group, peaking on day 63. Although the final EPG values were lower than those in the PC group (1525 ± 1083 EPG), the faecal egg counts were higher than those in the native 15-kDa protein group (436 ± 661 EPG). However, all treated groups showed decreases in their EPG values, whereas the PC group maintained its count until day 70 (*p* < 0.05) (Figure 2A).

### 3.2. iELISA

The data regarding the immune response after immunisation with the native *H. contortus* xL_3_ E/S protein and the S28 peptides, expressed as OD ± standard error, are shown in Figure 2C,D, respectively. The immune response was enhanced after the second immunisation (day 21) with the 15kDaNP, reaching the highest IgG responses on days 35 and 42 (0.796 ± 0.175 and 0.820 ± 0.212 OD, respectively). A decreased response was then observed until day 56 (0.629 ± 0.14 OD), with a slight increase thereafter, peaking on day 70 (0.740 ± 0.230 OD). The statistical analysis revealed differences with the PC group only on days 63 and 70 (*p* < 0.05). However, the NC group showed a lower immune response than the 15kDaNP group during the whole experiment. The OD ranged from 0.398 ± 0.078 to 0.666 ± 0.130.

In the S28P group, a marked increase in the OD value was observed after the first immunisation, peaking on day 35 (2.125 ± 0.880 OD). The OD value remained relatively constant until day 49, when a decrease was identified. The lowest response was observed on day 70 (1.509 ± 0.690 OD). By contrast, the NC and PC groups showed very similar low OD values throughout the whole experiment in comparison with the S28P group, although a slightly higher OD value in the PC than NC group was recorded from days 0 to 42 (*p* < 0.05). Interestingly, an opposite response was identified on day 70 (*p* < 0.05).

### 3.3. Adult Worm Counts and Morphometric Data

The 15kDaNP and S28P groups showed lower adult worm counts than the PC group, with an overall reduction of 72.28% and 65.21%, respectively (*p* < 0.05).

With respect to the worm measurements in the three groups, adult male and female parasites obtained from the S28P group showed a greater overall length and greater oesophageal length than those obtained from the PC and 15kDaNP groups (*p* < 0.05). Additionally, analysis of the female worms showed differences in the vulva–tail length, which was longer in the S28P group than in the PC and 15kDaNP groups (*p* < 0.05).

The oesophagus, spicules, tail width, and gubernaculum showed no significant differences among parasites recovered from the three experimental groups (*p* > 0.05) (Table 1).

### 3.4. Relative Gene Expression in Abomasal Tissue

**Figure 3:**
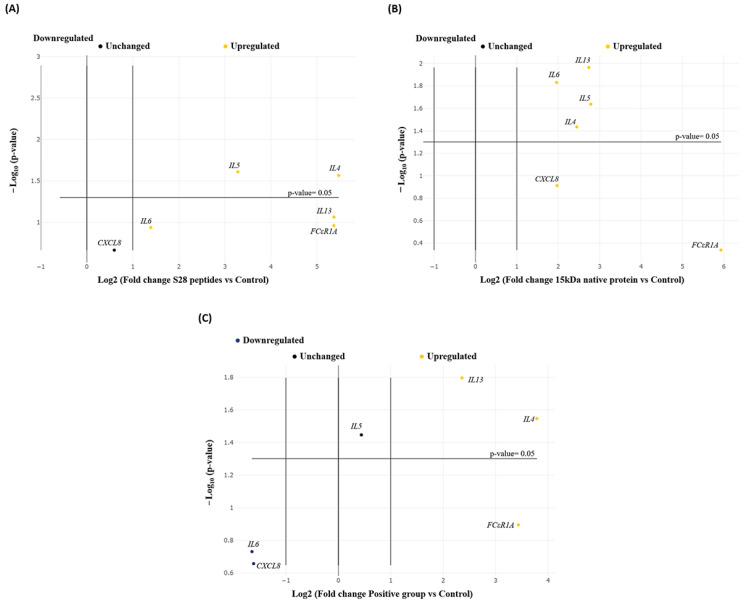
Dynamic of the expression levels of cytokine-immune genes. (**A**) S28 peptide group, (**B**) 15-kDa native protein group and (**C**) positive control group. RT-qPCR assays were performed in triplicates from three independent experiments. Differences were considered statistically significant when *p* < 0.05.

The S28P, 15kDaNP, and PC groups showed upregulation of the *IL4*, *IL5*, *IL13*, and *FCεR1A* genes in the abomasal tissue of the experimental lambs. The *IL5* expression values were statistically significant in all experimental groups, whereas *IL4* and *IL13* expression was statistically significant only in the 15kDaNP and PC groups. The *FCεR1A* gene upregulation was statistically significant only in the S28P group. Among the rest of the analysed genes, *IL6* and *CXCL8* were upregulated and downregulated in the 15kDaNP and PC groups, respectively, with significant overexpression of *IL6* in the 15kDaNP group (*p* < 0.05) (Table 2 and Figure 3).

### 3.5. Phenotypic and Genotypic Correlations

We assessed the correlation coefficients of the relative expression analysis of the immune response genes, as well as the IgG, haematocrit, and EPG values as phenotypic traits (Table S2). In the S28P group, *FCεR1A* showed a significant correlation with *IL5* and *IL6* (*p* < 0.05). Regarding the 15kDaNP group, significant and strong correlations were observed only among the cytokines evaluated (*p* < 0.05); *IL5* and *IL6* showed a correlation with *IL4*, while *CXCL8* did with *IL4* and *IL5*, and *IL13* did with *IL6*. Finally, the PC group showed a strong correlation only between the haematocrit level and the relative gene expression of *IL4* and *IL5* (*p* < 0.05).

## 4. Discussion

The negative effects of the use and abuse of chemical anthelmintic drugs required a search for alternative strategies, such as the use of immunising agents [41,42]. The overwhelming increase in the IgG values after the first immunisation in the S28P group with maintenance at a high level until day 63 provides evidence that these peptides are able to enhance a high and lasting IgG response in lambs. Although the IgG values were higher in the 15kDaNP than the PC group throughout the experiment, statistically significant differences were identified at the end of the experiment (day 70). Regarding the immunoprotective effect of S28P and 15kDaNP, both agents showed important reductions in the total adult *H. contortus* population recovered at necropsy (65% and 72%, respectively). These results are relevant because both antigens might be useful as immunoprotective agents against sheep haemonchosis. Recent studies have characterised diverse antigenic molecules derived from *Haemonchus* spp. E/S proteins using proteomic and genomic tools. These efforts have contributed to a better understanding of host–parasite interactions in the search for potential immunising agents [43,44]. Interestingly, humoral and cellular responses similar to those observed in the present study have also been reported in other studies that assessed antigens considered to be vaccine candidates [3,11]. Likewise, the *H. contortus* antigen Hc8, obtained from E/S proteins, reportedly led to a 50% reduction in the adult parasite count at necropsy in goats [45]. In another study, a group of researchers assessed the use of multiple peptides by recombinant technology in an effort to develop a vaccine against *Teladorsagia circumcincta*, a parasite prevalent mainly in temperate regions. The authors reported 55% to 75% reductions in the adult populations recovered at necropsy [45,46].

During the past few decades, the development of vaccines has demonstrated evidence of immunoprotection against parasitic nematodes in ruminants. Key antigens used in vaccines, such as the H11 and H-Gal-GP molecules found in the Barbervax vaccine [47,48,49], have shown promise in providing this protection. In these vaccines, IgG plays the most important role in conferring immunoprotection against haemonchosis. In the present study, we observed a rapid immune response of IgG in both immunised lamb groups, with the highest IgG value (*p* < 0.05) obtained for the S28P immunogen compared with the native E/S 15kDaNP group and PC group.

An increase in haematocrit levels was observed in animals treated with both 15kDaNP and S28P. This may have been due to the adult-worm reduction, particularly in the 15kDaNP group, leading to reduced invasion and tissue damage, with probably a more effective coagulation during blood intake by *Haemonchus contortus* [50]. On the other hand, the IgG response in the S28P group likely induced a reduction in *H. contortus* egg output with an aim to reduce the nematode infection, similar to the effects in the 15kDaNP and PC groups at the end of the experiment. In fact, several studies have evaluated the effect of E/S antigens in conferring protection through specific IgG, some of them with 14- and 24-kDa native E/S products from *H. contortus* [36,47,51]. The host immune mechanisms mediated by humoral and cellular responses have been found to modulate the damage caused by GIN infections in the early stages of host mucosal invasion [52,53]. E/S products involving proteins with enzymatic activity (i.e., metalloprotease, cysteine, serine, and aspartyl protease) are released during nematode invasion and feeding [54,55].

A previous immunological analysis of S28 peptides (from the serine protease family) and 15-kDa antigenic E/S proteins from *Haemonchus* sp. showed a high proliferation level of lymphocyte cells in in vitro assays with T_H_1 and T_H_2 upregulation of cytokine genes [28,29]. In the present study, both immunogens were able to reduce the establishment of adult nematodes, showing high activity of the *IL6* and *IL13* genes, as well as *CXCL8* associated with the inflammatory reaction. This effect against the nematodes can be considered the first host immune response induced by the mechanical pressure and enzymatic functions of the parasite, effectively reducing the invasion of the abomasal mucosa by the nematode larvae. The inflammatory reaction and the host immune response should be thoroughly analysed considering that parasitic helminths (*Fasciola* spp. and GINs) and protozoa (*Eimeria* spp.) of ruminants can provoke serious health problems and because the infected host may modulate the infection through the activity of *IL4*, *IL5*, *IL13*, and the *FCεR1A* receptor genes [56,57,58]. Studies measuring macrophage activity through *IL4*/*IL13* have demonstrated their anti-inflammatory function in regulating helminth infections. Additionally, these macrophages induce signals to maintain homeostasis and promote tissue repair from the injury caused by these parasites [59,60]. In the present study, the response of lambs immunised with the S28 peptides showed upregulation of the *IL4/IL13* and *FCεR1A* genes. We suggest that the defence response modulates the allergic and inflammatory reactions during the immunisation process. The results of the present study are important because the immune response demonstrated in these lambs can be used in future studies focused on preventing a high parasitic burden of *H. contortus* in lambs. The main challenge is identifying immunogenic antigens that can trigger an optimal immunoprotective response through immunological memory. A study using a recombinant IL-5 vaccine against *H. contortus* demonstrated the importance of eosinophils and confirmed the biological role played by IL-5 and eosinophils in the control of *H. contortus* in Canary hair sheep [61]. In addition, the authors noted extensive and significant differences in the lengths of the adult parasites between control and vaccinated sheep, showing longer parasites in the vaccinated group secondary to the inhibition of IL-5 and eosinophils. The study confirmed the function of the *IL5* gene as a defender against parasitic helminths such as *H. contortus.* In the present study, we found differences in the size of the parasites in both immunised groups relative to those recovered from the PC group.

Interestingly, the adult parasites recovered from the S28P group were longer than those recovered from the PC group. Previous studies comparing sheep with high and low immune responses against GINs revealed a relationship of IgA and IgE with susceptibility and resistance traits to GINs. These studies evaluated their association with worm size and female fecundity, finding that worm size increased in susceptible sheep, which might be related to nematode survival [62,63,64].

In this study, the immunogenic activity of S28 peptides from *H. contortus* appeared to be associated with the increasing size and length of the body of the adult parasites, as well as the egg output as observed in the IL- and IgG-related responses. However, further studies are necessary to confirm this phenomenon. By contrast, a negative correlation of 15kDaNP was observed between the phenotype traits and the cytokine genes. Likewise, *IL-6* and *IL-13* were found to be upregulated, confirming the inflammatory reaction induced by the 15-kDa native antigen. Maza-Lopez et al. (2021) [28] identified a strong enhancer of inflammatory cells and T_H_2 cell types that modulated the infection. Finally, the study of these two immunogens derived from *Haemonchus* proteins has revealed how the host–nematode interrelationship induces immunoprotection in grazing hosts. The use of S28P and 15kDaNP can be explored as potential vaccines against sheep haemonchosis in tropical and subtropical regions. Nevertheless, more studies are needed which have higher numbers of animals, a longer experimental duration, and that use other adjuvants. An assay of the dose/response and frequency of treatments with these antigens would be helpful to determine whether the immunoprotective effect can be enhanced.

## 5. Conclusions

The study of a semi-purified protein (15-kDa) and a mix of two synthetic peptides (S28) derived from *Haemonchus contortus*, one of the most important parasites in small ruminants, exposed the efficacy of inducing an immune response during haemonchosis, reducing the nematode infection. However, further studies are necessary to increase the knowledge about the potential of these E/S proteins and synthetic peptides as possible vaccine candidates in grazing animals.

## Figures and Tables

**Table 1:** Total adult *Haemonchus contortus* (males and females) recovered at necropsy of three experimental groups of lambs, morphological measurements, and reduction percentages.

Group	Female (Mean ± SE) (**)					Male (Mean ± SE) (**)					Total Worm Count	Reduction %
	Worm Count	Total Length	Vulva Tail	Oesoph.	Width Tail	Worm Count	Total Length	Spicules	Oesoph.	Gubern.		
15kDaNP	283 *	11,558.73 ± 601.78	2027.73 ± 117.56	1105.05 ± 18.77 *	85.59 ± 16.15	226 *	8430.94 ± 334.67	425.73 ± 4.75	1057.35 ± 21.74	211.10 ± 3.44	509 *	72.28
S28P	356 *	14,270.68 ± 383.11 *	2502.92 ± 75.62 *	1210.23 ± 21.33	82.64 ± 3.40	283 *	10,465.53 ± 211.55 *	434.02 ± 4.64	1795.84 ± 670.69	210.35± 3.54	638 *	65.21
PC	1053	11,609.55 ± 512.61	2103.51 ± 73.92	1197.01 ± 27.18	89.15 ± 3.41	783	8940.49 ± 328.03	434.75 ± 3.38	1098.86 ± 34.98	214.84 ± 3.50	1835	

** 25 adult females and males were selected for morphological characterization; data measurements: µm, * *p* < 0.05. Oesoph = oesophagus; Gubern = gubernaculum.

**Table 2:** Relative expression levels of cytokine-immune genes from abomasal tissue from experimental groups challenged with *H. contortus* compared with negative control group.

Gene	15kDaNP	S28p	Positive Group
*IL4*	5.46 *	41.27	13.81 *
*IL5*	6.90 *	9.73 *	1.36 *
*IL6*	3.89 *	2.62	−0.32
*CXCL8*	3.92	1.51	−0.33
*IL13*	6.67 *	41.38	5.13 *
*FCεR1A*	61.02	44.38 *	10.82

*IL*: interleukin; *CXCL8*: C-X-C motif chemokine ligand 8; *FCεR1A*: IgE receptor. Differences were considered statistically significant when *p* < 0.05. Bold black number = upregulated expression level; minus sign and black number = downregulated expression level; * *p* ≤ 0.05.

## Data Availability

Data are contained within the article and Supplementary Materials.

## Supplementary Materials

The following supporting information can be downloaded at: https://www.mdpi.com/article/10.3390/pathogens13070604/s1, Table S1: access numbers and dissociation temperatures of evaluated genes in the relative expression analysis; Table S2: correlation (r2) between phenotypic traits and immune genes in the positive control group and lambs immunised with *H. contortus* antigens. The analysed results correspond to the data at the end of the experimental evaluation.
