# Supplementary material for: Excreted/Secreted 15-kDa Proteins and Serine Protease Peptides from Haemonchus contortus Act as Immune-Response Enhancers in Lambs
Source: Pathogens. 2024 Jul 22;13(7):604. doi: 10.3390/pathogens13070604 (PMC11280362; doi:10.3390/pathogens13070604)
Supplement: Supplementary file 1 [file pathogens-13-00604-s001.zip › pathogens-3117683-supplementary.pdf]

Supplementary Table S1. Access numbers and dissociation temperatures of evaluated genes in the relative expression analysis

Genes	Melting temperature (Tm °C)	GenBank Access N°
IL4	80.6	NM_173921
IL5	76.68	NM_173922
IL6	81.5	NM_173923
IL8	80.5	NM_173925
IL13	79.9	NM_174089.1
FCεR1A	81.8	NM_001100310
β-actin	84.25	XM_027528015.1
GAPDH	85.3	NM_001190390.1

Supplementary Table S2. Correlation (r2) between phenotypic traits and immune genes in the positive control group and lambs immunised with *H. contortus* antigens. The analysed results correspond to the data at the end of the experimental evaluation.

GROUPS		*IL5*	*IL6*	*CXCL8*	*IL13*	*FCεR1A*	**EPG**	**Ht**	**IgG-15**	**IgG-S28**
	IL4	-0.274	-0.175	-0.211	-0.236	-0.262	-0.342	0.363		-0.216
	IL5	1	0.579	0.688	-0.273	.905*	-0.498	0.446		0.709
	IL6		1	-0.174	-0.074	.870*	-0.304	-0.065		0.063
	CXCL8			1	-0.313	0.319	-0.283	0.476		0.768
**S28P**	***IL13***				1	-0.174	0.805	-0.436		0.267
	FCεR1A					1	-0.450	0.245		0.474
	**EPG**						1	-0.797		0.034
	**Ht**							1		0.324
	**IgG**									1
	IL4	.848*	.889**	.902**	0.686	-0.005	-0.445	0.088	-0.416	
	IL5	1	0.601	.951**	0.317	0.211	-0.492	0.199	-0.301	
	IL6		1	0.615	.914**	-0.019	-0.481	0.233	-0.471	
	CXCL8			1	0.325	0.005	-0.378	-0.010	-0.328	
**15kDaNP**	***IL13***				1	0.104	-0.474	0.163	-0.389	
	FCεR1A					1	-0.315	0.396	0.316	
	**EPG**						1	-0.547	-0.229	
	**Ht**							1	0.432	
	**IgG**								1	
	IL4	1.000**	0.685	0.683	0.144	0.682	-0.528	.939*	0,823	-0,473
**PC**	***IL5***	1	0.675	0.673	0.121	0.672	-0.541	.944*	0,829	-0,457
	IL6		1	1.000**	0.346	1.000**	-0.568	0.591	0,154	-.884*
	CXCL8			1	0.348	1.000**	-0.569	0.589	0,151	-.883*

IL13	1	0.368	-0.042	-0.194	-0,107	-0,224
FCεR1A		1	-0.562	0.581	0,149	-.883*
**EPG**			1	-0.459	-0,223	0,156
**Ht**				1	0,830	-0,459
**IgG-15**					1	0,009
**IgG-S28**						1

**p ≤ 0.01; *p ≤ 0.05.
